# Association Between a Decrease in Blood Monocyte Counts and Necrotizing Enterocolitis in Preterm Infants

**DOI:** 10.7759/cureus.84906

**Published:** 2025-05-27

**Authors:** Supriya Bisht, Kim Roger, Sharef Al-Mulaabed, Fernanda E Kupferman

**Affiliations:** 1 Department of Pediatrics, Division of Newborn Medicine, University of Mississippi Medical Center, Jackson, USA; 2 Department of Pediatrics, Division of Neonatology, One Brooklyn Health - Brookdale Hospital Medical Center, Brooklyn, USA; 3 Division of Pediatric Gastroenterology, Orlando Health Arnold Palmer Hospital for Children, Orlando, USA

**Keywords:** absolute monocyte count, feeding intolerance, necrotizing enterocolitis, neonate, preterm

## Abstract

Objective: To explore the association between absolute monocyte count (AMC) and necrotizing enterocolitis (NEC) in preterm neonates and to assess whether a fall in AMC can be used as a biomarker to predict an increased risk for NEC.

Study design: This was a retrospective study of preterm neonates with NEC or a first episode of feeding intolerance (FI). Their complete blood count (CBC) was evaluated for AMC at three time points, first at baseline, second at the onset of NEC/FI, and third afterwards. Statistical analysis was done using Student's t-test and the chi-squared test. The p-value of <0.05 was considered significant.

Results: Of the total 92 newborns, 29 (31.5%) had NEC, while 63 (68.5%) had FI. NEC and FI groups were comparable in gestational age (GA) and birth weight. There was a significant increase in AMC in the FI group (p<0.001) from baseline to the onset of FI. However, the AMC had a fall in the NEC group from baseline to the onset of NEC, which was not statistically significant (p=0.074).

Conclusion: A decrease in AMC was associated with the presence of NEC. A fall in the AMC could be a biomarker to identify an increased risk of NEC in preterm neonates with feeding problems.

## Introduction

Necrotizing enterocolitis (NEC) is the leading cause of morbidity and mortality in preterm neonates, which often presents as a catastrophic gastrointestinal (GI) emergency [1,2]. It occurs in approximately 4%-11% of all very low birth weight preterm (VLBW) infants [2]. The initial clinical symptoms can be abdominal distension, bloody stools, and feeding intolerance (FI). The presence of pneumatosis intestinalis, portal venous gas on abdominal radiography, is the pathognomonic finding of NEC [3]. The intestinal inflammation and coagulative necrosis [4] is the result of an ongoing intense inflammatory response with leukocyte infiltrate in NEC [2].

On the other side, FI is significantly common in premature infants and is usually described as the presence of significant gastric residuals (more than one-third of feeds), biliary or bloody vomiting, and/or abdominal distention. FI may be benign or an initial presentation of subsequent NEC [5,6]. Early diagnosis and management of NEC is critical but challenging due to a lack of reliable biomarkers [4]. Timely diagnosis can also facilitate early treatment of NEC, including possible need for transporting infants with NEC to NICU with higher facilities providing surgical care, since advanced NEC can rapidly progress to severe bowel necrosis and bowel perforation, which require surgical intervention [7]. There have been efforts to look for early markers of NEC in the complete blood count (CBC), one of which is the absolute monocyte count (AMC).

Monocytes are an integral part of the reticuloendothelial system, which briefly stay in the blood, followed by transmigration to tissues to become mononuclear phagocytes. Blood monocytes also increase with gestational age (GA) [8]. The total blood monocyte pool is distributed as a circulating and a marginating pool [9]. In NEC, the migration of circulating monocytes into intestinal tissue can cause a sudden decrease in peripheral blood monocyte counts [4]. Other studies suggested that AMC change can help identify NEC along with prognostication of the severity of NEC [10,11]. However, further research and studies are required to identify the relationship between a fall in blood monocyte count and NEC.

The primary aim of this study was to explore the association between AMC and NEC among preterm infants and evaluate its potential as a biomarker to predict increased risk for NEC. The secondary aim was to evaluate other parameters in the CBC and observe if there are any changes associated with NEC.

## Materials and methods

Selection of participants

This was a retrospective case-control study of preterm neonates (born <37 week gestation age) with feeding concern who had either been diagnosed with NEC stage II (definite NEC) to stage III (advanced NEC) as per the modified Bell’s criteria [7] or had developed first episode of FI (never diagnosed as NEC) during their admission at the NICU at One Brooklyn Health at Brookdale Hospital Medical Center from November 2013 to May 2020. The preterm neonates who did not have a CBC in the preceding seven days before developing NEC or FI or after it, infants with genetic syndrome (trisomy 13, 18, 21), who have severe congenital anomaly involving the GI tract, or infants with any prior GI surgery or transfer out were excluded. This study was approved by the institutional review board.

Data collection and measurement

Infant’s CBC parameters, which included AMC, were obtained from electronic health records. Three sets of values were obtained -the first one is the baseline CBC (within seven days before the feeding problem of NEC/FI); the second one was at the onset of NEC/FI, which is the day an infant was made nil per oral (NPO) due to clinical concerns; and the third one was post NEC/FI and was considered up to seven days after the onset of NEC/FI. Additionally, demographic and clinical data of the infants was obtained, including maternal characteristics, formula feeds, presence of umbilical venous and arterial lines, presence of respiratory distress syndrome (RDS), culture positive sepsis, packed red blood cell (PRBC) transfusion, shock requiring inotropes preceding the NEC/FI episode, presence of patent ductus arteriosus (PDA) treatment, intraventricular hemorrhage (IVH), day of developing NEC/FI, and duration of NICU stay.

Statistics

Statistical analysis was done using the Statistical Product and Service Solutions (SPSS, version 20; IBM SPSS Statistics for Windows, Armonk, NY) software. The mean (± standard deviation) or median (interquartile range) was used to present the numerical data, as appropriate. Differences in means of continuous variables between the two groups were analyzed using Student's t-test, while differences in the categorical variables were assessed using the chi-square test. The paired Student's t-test was used to analyze the changes in laboratory data at different timepoints, in each group. A P-value of <0.05 was used as a cut-off for all tests of statistical significance.

## Results

**Figure 1:**
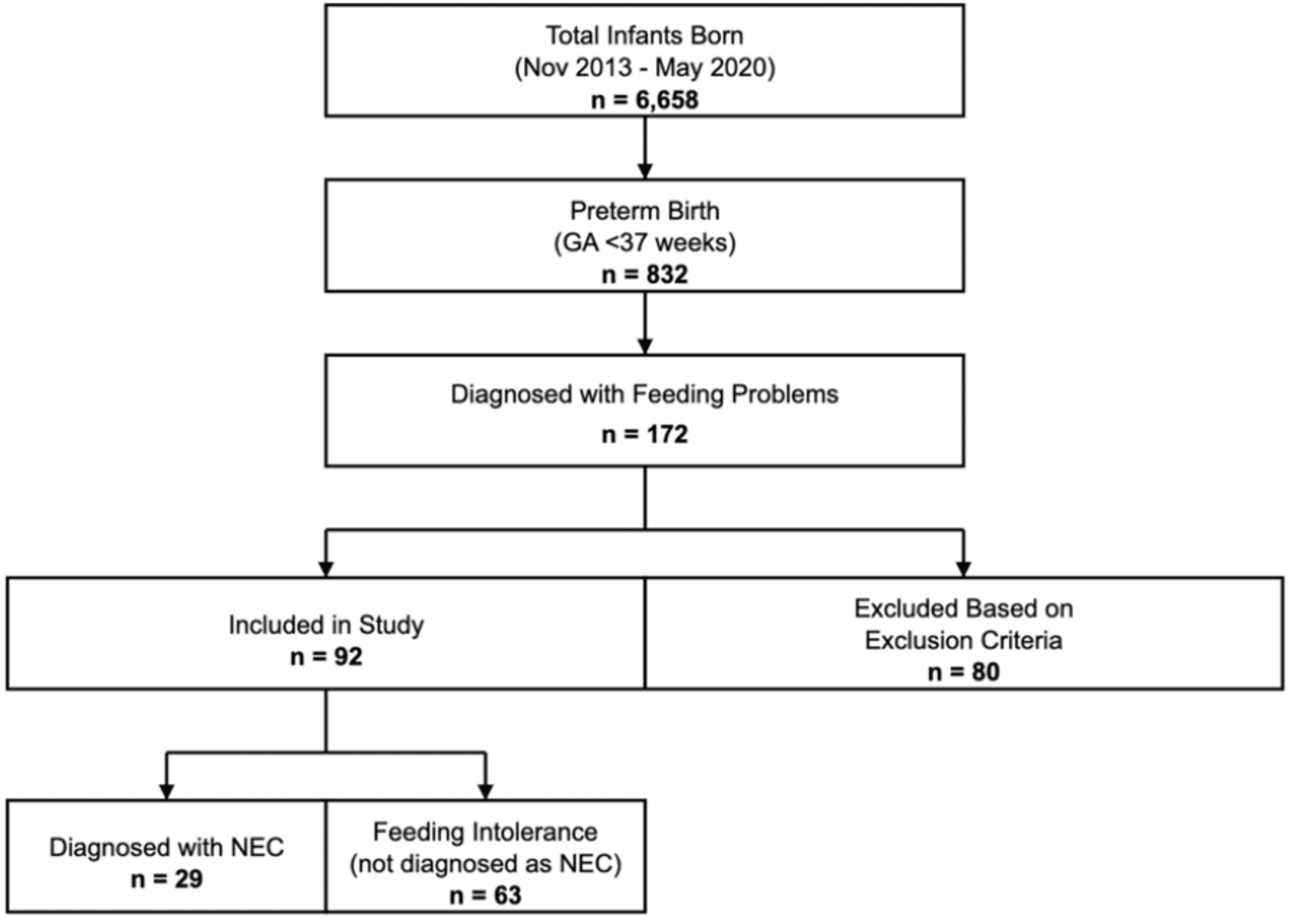
Flow diagram of the subjects in the study. GA = Gestational age; NEC = Necrotizing enterocolitis

A total of 6,658 infants were born between November 2013 and May 2020, as depicted in Figure 1.

A total of 832 subjects were born preterm, with gestational age (GA) <37 weeks. One hundred and seventy-two infants were diagnosed with feeding problems, of which 92 newborns were included in the study and 80 were excluded based on the exclusion criteria. Among 92 included infants, 29 (31.5%) had NEC, while 63 (68.5%) had FI (not diagnosed as NEC). In the NEC group, 22 cases (75.9%) were NEC stage II, while seven cases were NEC stage III (24.1%). Infants in the NEC and FI groups were similar in the majority of baseline clinical characteristics (Table 1).

**Table 1:** Demographic and baseline data of neonates with feeding problem (FI/NEC), n=92). SD = Standard deviation, IQR = Interquartile ratio; FI/NEC: Feeding intolerance/necrotizing enterocolitis; PRBC = Packed red blood cells ^1^ Comparison was done using an independent t-test. ^2^ Comparison was done using the chi-square test. ^3 ^Comparison was done using the Mann-Whitney test.

Characteristics	FI (n = 63)	NEC (n=29)	Statistical value	P value
Gestational Age (Weeks), Mean (±SD)	29.2 (±3.5)	29.1 (±4.3)	t = - 0.089	0.929^ 1^
Birth Weight (Grams), Mean (±SD)	1233 (±518)	1330 (±679)	t = 0.750	0.455^ 1^
Gender (Male), n (%)	31 (49%)	15 (52%)	X^2^ = 0.050	0.822^ 2^
Maternal Race (Black), n (%)	53 (84%)	23 (79%)	X^2^ = 1.093	0.779^ 2^
Caesarian Section Delivery, n (%)	42 (67%)	11 (38%)	X^2^ = 6.715	0.010^ 2^
Maternal Chorioamnionitis, n (%)	6 (10%)	24 (7%)	X^2^ = 3.495	0.062^ 2^
GBS/Cervical Culture Positive, n (%)	4 (6%)	5 (17%)	X^2^ = 2.670	0.102^ 2^
Maternal Treatment for GBS Positive or Unknown, n (%)	35 (56%)	16 (55%)	X^2^ =327	0.849^ 2^
Antenatal Steroids Received, n (%)	49 (78%)	19 (66%)	X^2^ = 1.548	0.213^ 2^
Maternal Urine Drug Test Positive, n (%)	7 (11%)	7 (24%)	X^2^ = 2.612	0.106^ 2^
APGAR Score at 5 min, Median (IQR)	8 (6-9)	8 (7-9)	U = 820	0.580^ 3^
Formula Feeds, n (%)	42 (67%)	19 (66%)	X^2^ = 0.012	0.914^ 2^
Blood Culture Positive Sepsis, n (%)	11 (18%)	9 (31%)	X^2^ = 2.373	0.305^ 2^
Received PRBC Transfusion, n (%)	34 (54%)	19 (66%)	X^2^ = 1.085	0.298^ 2^
Umbilical Artery Catheter, n (%)	36 (57%)	17 (59%)	X^2^ = 0.018	0.894^ 2^
Umbilical Vein Catheter, n (%)	40 (64%)	20 (69%)	X^2^ = 2.733	0.255^ 2^
Requirement of Inotropes, n (%)	11 (18%)	5 (17%)	X^2^ = 0.001	0.979^ 2^
Patent Ductus Arteriosus, n (%)	25 (40%)	15 (52%)	X^2^ = 1.172	0.279^ 2^
Received PDA Treatment, n (%)	6 (18%)	7 (47%)	X^2^ = 4.237	0.040^ 2^
Intraventricular Hemorrhage, n (%)	6 (6%)	6 (21%)	X^2^ = 4.215	0.040^ 2^
Respiratory Distress Syndrome, n (%)	53 (84%)	23 (79%)	X^2^ = 0.321	0.571^ 2^
Duration of Stay in Days, Mean (±SD)	63 (±39)	66 (±37)	t = 0.252	0.801^ 1^
Day of Feeding Problem (NEC/FI), Mean (±SD)	19 (±13)	16 (±14)	t = - 0.995	0.323^ 1^

The infants in the NEC group were born at a mean (±SD) GA: 29.1 (±4.3) weeks. Similar to it, the infants in the FI group were born at a mean (±SD) GA: 29.2 ±3.5 weeks. The birth weight was also similar, with mean (±SD) birth weight in the NEC group being 1,330 (±679) g, and in the FI group mean (±SD) birth weight was 1233 ±518 g. The differences observed between the two groups were notable that the FI group had more infants born by caesarean delivery, while the NEC group had more infants with PDA treatment and IVH.

Tables 2-3 present the CBC parameters in each group at the three time points (before, during, and after the FI/NEC event).

**Table 2:** CBC values at baseline, at onset of FI/(NPO) and afterwards (n=63). Note: All comparisons were done using a paired t-test. ^1^ Comparison from baseline to the onset of FI. ^2^ Comparison between the onset of FI and afterwards CBC = Complete blood count; FI/(NPO = Feeding intolerance/nil per oral

Characteristics		Mean (±SD)	Statistical value	P value
White Blood Count (WBC/microliter	Baseline	13,957±6899	t = -2.144	0.036 ^1^
	Onset of FI	15,647±6907	-	-
	Afterwards	15,104±6275	t = 0.857	0.395 ^2^
Platelet count x 10^9^/liter	Baseline	262±134	t = -3.133	0.003 ^1^
	Onset of FI	300±128	-	-
	Afterwards	301±107	t = -0.082	0.935 ^2^
Absolute Monocyte Count (AMC)/microliter	Baseline	1673±1287	t = -4.295	<0.001 ^1^
	Onset of FI	2397±1420	-	-
	Afterwards	2027±1392	t = 1.937	0.057 ^2^
Absolute Neutrophil Count (ANC)/microliter	Baseline	5869±4120	t = -0.418	0.678 ^1^
	Onset of FI	6082±3802	-	-
	Afterwards	5532±4112	t = 1.022	0.311 ^2^
Absolute Lymphocyte Count (ALC)/microliter	Baseline	4816±2367	t = -1.308	0.196 ^1^
	Onset of FI	5189±2200	-	-
	Afterwards	5290±2117	t = -0.350	0.728 ^2^
Hemoglobin gm/deciliter	Baseline	13.5±2.6	t = 2.301	0.025 ^1^
	Onset of FI	12.9±2.5	-	-
	Afterwards	12.3±2.2	t = 2.154	0.035 ^2^

**Table 3:** CBC values at baseline, at onset of NEC/(NPO), and afterwards (n=29). Note: All comparisons were done using a paired t-test. ^1^ Comparison from baseline to the onset of necrotizing enterocolitis (NEC). ^2^ Comparison between the onset of NEC and afterwards. CBC = Complete blood count

Characteristics		Mean (±SD)	Statistical value	P value
White blood count (WBC) /microliter	Baseline	16,427±8302	t = 1.856	0.073 ^1^
	Onset of NEC	14,237±7910	-	-
	Afterwards	16,844±7449	t = - 1.660	0.030 ^2^
Platelets x 10^9^/liter	Baseline	270±131	t = - 0.701	0.489 ^1^
	Onset of NEC	285±144	-	-
	Afterwards	282±145	t = 0.152	0.880 ^2^
Absolute Monocyte Count (AMC)/microliter	Baseline	2539±1859	t = 1.855	0.074 ^1^
	Onset of NEC	2057±1670	-	-
	Afterwards	2610±1366	t = -1.488	0.107 ^2^
Absolute Neutrophil Count (ANC)/microliter	Baseline	7687±5631	t = 0.765	0.451 ^1^
	Onset of NEC	7126±5611	-	-
	Afterwards	6717±4962	t = 0.385	0.703 ^2^
Absolute Lymphocyte Count (ALC)/microliter	Baseline	4529±2132	t = 0.953	0.358 ^1^
	Onset of NEC	4045±2136	-	-
	Afterwards	4702±2368	t = -1.326	0.195 ^2^
Hemoglobin gm/deciliter	Baseline	13.7±3.3	t = 1.020	0.358 ^1^
	Onset of NEC	13.2±3.1	-	-
	Afterwards	12.3±2.2	t = 1.651	0.195 ^2^

**Figure 2:**
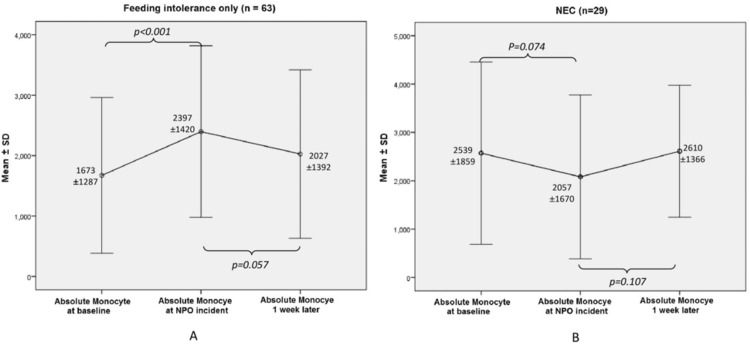
The changes of absolute monocyte count (AMC) at three time points in FI and NEC groups (A) Feeding intolerance (FI) group. (B) Necrotizing enterocolitis (NEC) group

There was a significant increase in white blood cell (WBC) count, from baseline to the onset of feeding problem in the FI group (p=0.036). The NEC group had an initial decrease in WBC count from baseline to the onset of NEC (p=0.073) with a trend towards statistical significance. In addition, a significant rise in WBC from the onset of NEC to post-NEC (p=0.030) was observed. Results in Table 2 also suggested a significant increase in AMC in the FI group from baseline to the onset of FI (p<0.001), whereas in the NEC group (Table 3), the AMC had a fall from baseline to onset of NEC (p=0.074), showing a trend for decrease in AMC. The changes of AMC in each group are further depicted in Figure 2.

The platelet counts showed a significant increase, from baseline to the onset of the feeding problem in the FI group (p=0.03), as shown in Table 2. No significant difference was observed in the NEC group. The hemoglobin level was noticed to have fallen significantly in the FI group, with no significant changes in the NEC group.

Table 4 shows the comparison of changes in CBC parameters between the FI and NEC groups.

**Table 4:** Comparison between FI and NEC groups, in changes of CBC at three time points (n=92). ^1^ Comparison was done using the independent t-test. ^2^ Comparison was done using the Mann-Whitney test All data are expressed as mean ±SD except as indicated. * per microlit, ** x 10^9^ per lit, *** gm per dL CBC = Complete blood count; Hb = Hemoglobin; FI = Feeding intolerance; IQR = Interquartile range; NEC = Necrotizing enterocolitis; WBC = White blood count

Characteristics		FI (n = 63)	NEC (n=29)	Statistical value	P value
Change from baseline to onset of NEC/FI	WBC*, Mean (±SD)	1690±6259	-2190±6352	t = -2.750	0.007 ^1^
	Platelet**, Mean (±SD)	39±98	15±116	t = -1.010	0.348 ^1^
	Absolute Monocyte Count*, Mean (±SD)	724±1338	-482±1401	t = -3.960	<0.001 ^1^
	Absolute Neutrophil count*, Mean (±SD)	213±4054	-561±3951	t = -0.858	0.393 ^1^
	Absolute Lymphocyte count*, median (IQR)	556 (-986 to 1830)	-984 (-2325 to 1532)	U = -1.479	0.137 ^2^
	Hb***, median (IQR)	-0.8 (-1.7 to 0.8)	-1 (-2.5 to 1.3)	U = -0.179	0.765 ^2^
Change from onset of NEC/FI to lab parameter afterwards	WBC*, Mean (±SD)	-543±5026	2607±8455	t = 2.229	0.028 ^1^
	Platelet**, Mean (±SD)	1±89	-3±108	t =-0.185	0.854 ^1^
	Absolute Monocyte Count*, Mean (±SD)	-370±1517	467±1689	t = 2.372	0.020 ^1^
	Absolute Neutrophil Count*, Mean (±SD)	-550±4274	-409±5721	t = 0.0132	0.895 ^1^
	Absolute Lymphocyte Count*, Median (IQR)	333 (-1582 to 1574)	828 (-1753 to 2354)	U = -1.479	0.375 ^2^
	Hb***, Median (IQR)	-0.7 (-1.9 to 0.7)	-0.5 (-3 to 1.4)	U = -0.336	0.737 ^2^

There was a significant difference in the change of WBC and AMC, both from baseline to onset of NEC/FI (p=0.007 and p<0.001, respectively) and from onset of NEC/FI to values afterwards (p=0.028 and p=0.020, respectively).

## Discussion

This study focuses on the potential role of AMC as a biomarker for NEC in preterm neonates. Our findings demonstrate a trend towards a decrease in AMC in neonates with NEC. Although the decrease was statistically not significant, the trend of fall in AMC with the onset of NEC is opposite to that observed in FI, in which a significant increase in AMC was observed, and is considered a crucial finding. Further research with an increased number of subjects is suggested for significant results.

These results are still noteworthy in the context of previous studies, indicating that a reduction in peripheral blood monocyte counts is observed in NEC [4,10-12]. This is consistent with the hypothesis that NEC involves a robust inflammatory response characterized by monocyte infiltration into the gut. In contrast, the increase in AMC in FI reflects a different inflammatory process. This distinction highlights the potential utility of AMC as a diagnostic marker for NEC, particularly in the early stages when clinical symptoms often overlap with FI.

While no studies explicitly report an increase in AMC in FI, the milder inflammatory response associated with FI may result in the elevated monocyte counts [11]. Early identification of NEC is critical, as delayed diagnosis can lead to severe complications of advanced NEC, including bowel perforation, sepsis, and increased mortality [2]. The pathognomonic radiographic signs, which are diagnostic of NEC, do not appear early when the infant starts showing initial symptoms of NEC. It is a usual norm in NICUs to do a CBC and other supporting investigations if there is a change in the clinical status of an infant. The trend of fall in AMC in NEC compared to an increase in FI can provide a helpful insight about the possibility of NEC in preterm infants who present with GI symptoms. It is quite feasible to look for AMC during CBC evaluation without added cost or new investigation [4].

The trend in WBC from initial nonsignificant leucopenia from baseline to the onset of NEC, followed by leucocytosis post-diagnosis of NEC, may reflect disease progression and associated immune dysregulation. Most patients with NEC develop leukocytosis and neutrophilia [2]. Studies suggest that thrombocytopenia, lymphopenia, neutropenia, and leukopenia are observed in fulminant NEC [13]. While our study did not suggest any significant changes in platelet count, lymphocyte count, and neutrophil count, our NEC group was neither subdivided nor analyzed for the severity of NEC, which might have suggested the findings. The drop in hemoglobin in FI might be a coincidental finding, while the NEC group had stable hemoglobin, which could reflect possible PRBC transfusion, as our study did not collect data on PRBC transfusion after developing NEC/FI.

Table 4 highlights a significant difference in the changes of WBC and AMC, both from baseline to onset of NEC/FI (p=0.007, p<0.001), and from onset of NEC/FI to values afterwards (p=0.028, p=0.020), which further signifies the association between the fall in AMC and NEC onset.

Our FI and NEC groups were almost similar in clinical characteristics, with the exception of more infants delivered by cesarean section in the FI group. The NEC group also had a higher number of infants receiving PDA treatment, which is similar to other studies that resulted in increased risk of NEC in preterm infants receiving medical treatment (indomethacin) for PDA [14], and the presence of PDA being associated with an increased risk of mortality in NEC [15]. The NEC group had more IVH compared to the FI group, which is similar to other studies, which suggest that infants with surgical NEC had an increased association with the development of IVH [16].

Despite these promising results, our study has many limitations. Our study is a single-center and retrospective, which limits the findings. Additionally, the exclusion of neonates without a CBC count may introduce selection bias. We did not subdivide the NEC group further into NEC stages II (definite NEC) and III (advanced NEC), which could have suggested any further trend in AMC with the severity of NEC. We did not grade the change of AMC values. Future prospective multicenter studies with larger sample sizes are recommended to validate these findings.

## Conclusions

Our study suggested that a fall in the AMC can serve as a useful biomarker to identify an increased risk of NEC in preterm neonates who present with feeding problems. The decrease in AMC in NEC, coupled with a significantly elevated AMC in FI, underscores the distinct inflammatory responses in these conditions. Additionally, the significant difference in the changes of AMC further indicates the association between the fall in the AMC and NEC onset. Our research contributes important insights into the pathophysiology of neonatal gastrointestinal disorders and supports the growing evidence for hematological markers in NEC diagnosis. This could serve as an added benefit with no additional cost to facilitate clinicians for early diagnosis of NEC in preterm infants, particularly in the early stages when clinical symptoms often overlap with FI.
